# The influence of bilingual experience on executive function under emotional interference: Evidence from the N1 component

**DOI:** 10.3389/fpsyg.2023.1107994

**Published:** 2023-03-31

**Authors:** Yachen Tao, Zhi Zhu, Yan Liu

**Affiliations:** ^1^Bristol Business School, University of the West of England, Bristol, United Kingdom; ^2^Faculty of Education, Yunnan Normal University, Kunming, China; ^3^Faculty of Foreign Languages and Cultures, Kunming University of Science and Technology, Kunming, China

**Keywords:** bilingual experience, executive function, emotional interference, ERP technology, inhibition control

## Abstract

The influence of bilingual education and experience on an individual’s information-processing ability has recently been a hot issue in international studies. Previous studies have found that bilingual experience affects executive function, but the results remain controversial. Executive function refers to the conscious control of purposeful behavior. It is responsible for processing high-level action controls, including such sub-functions as inhibitory control, cognitive switching, and working memory updating. Emotion, as an essential factor in daily life, also has a complex interaction with executive function. This paper explores whether the bilingual cognitive advantage effect can continue in the more complex conditions of emotional interference. To investigate the specific electrophysiological characteristics of the participants at different stages of cognitive processing, we used a combination of the behavioral and ERP experiments in which the positive, neutral, and negative emotional stimuli were selected as emotional interference conditions and the emotional Simon paradigm, the cognitive switching of emotion paradigm, and the emotional N-back paradigm was adopted. The results show that the main effect of the N1 component amplitude is significant. Specifically, the amplitude of the N1 component in the proficient bilinguals is significantly smaller than that of the non-proficient bilinguals, while the main effects of other component groups are not significant, indicating that under the condition of emotional interference, the influence of bilingual experience on executive function only exists in the early attention stage and that the bilingual experience can improve the individual’s attentional control and speed up attention processing in the early attention stage.

## 1. Introduction

Bilinguals are those who master two languages (Nagel et al., 2015). According to the proficiency level of bilingual experience, bilinguals can be proficient and non-proficient bilinguals. Proficient bilinguals refer to individuals with equal proficiency in the two languages, while non-proficient bilinguals are those whose proficiency in the second language is lower than that of the first language (Gong et al., 2002). Bilingual experience is a high-intensity experience that affects an individual’s brain function and cognitive activity (Bialystok, 2011). As Antoniou (2019) found, bilingual experiences could bring brain changes and improve cognitive aging. Some researchers have found that learning a second language increases the density of gray matter in the left sub-parietal cortex, and second language proficiency is significantly associated with neurophysiological changes. That is, the higher the second language proficiency and the younger the age of acquisition, the more pronounced the change is (Mechelli et al., 2004). In addition, studies of participants whose mother tongue was English and their second language was Spanish, French, or German found that bilingual experiences affected the plasticity of brain structures and the brain’s cognitive function (Miyake and Friedman, 2012). This influence includes not only speech-related cognitive functions, such as inhibition of extraneous distracting information in reading (Bialystok, 2009; Blumenfeld and Marian, 2011), metalinguistic ability (Bialystok, 2007); it can also affect nonverbal cognitive functions, such as executive function (Kroll and Bialystok, 2013; Nichols et al., 2020).

Executive function is an essential cognitive function in goal-oriented behavioral control and self-control, closely related to individuals’ social and cognitive development (Hughes and Ensor, 2007; Best et al., 2009). Executive functions generally include inhibitory control, cognitive switching, and working memory updating (Miyake and Friedman, 2012). Inhibitory control is commonly expressed as an ability to select and store task-related information during task processing and ignore interfering information (Botvinick et al., 2001). Cognitive switching, the ability to transform mental representations and adapt to changing or unpredictable situations, namely, the ability to shift between different tasks with varied requirements and rule patterns, is an essential feature of human intelligence (Tannock and Schachar, 1995). Working memory updating is a process of memory trade-in in a central executive system, i.e., constantly updating temporarily stored information (Morris and Jones, 1990). As seen from the above, previous studies on the impact of bilingual experience on executive function have generally carried out specific research on the sub-functions of the above three executive functions.

The earliest evidence of the effect of bilingual experience on executive function comes from inhibitory control, and it is argued that bilingual experience can improve an individual’s inhibitory control. Researchers using Stroop color-naming tasks have found that bilinguals have an advantage over monolinguals in recognizing font colors (Coderre and Van Heuven, 2014). Bialystok et al. (2008) used the Stroop paradigm to study young participants and found that bilinguals had a more negligible Stroop effect than monolinguals. Young bilinguals in the Simon paradigm responded faster to conflict tasks than monolinguals. Similarly, the Flanker paradigm was used to obtain speedier task responses in bilinguals than monolinguals in middle-aged participants (Emmorey et al., 2008). Additionally, Bilingual experience can affect individual cognitive switching. Some researchers have found that low-proficiency L2 learners experienced significantly higher switching costs in simple language switching tasks than in complex language switching tasks (Calabria et al., 2012). Prior and Macwhinney (2010) conducted a cue-switching task among college students and found that bilinguals had an advantageous effect on cue-switching ability. Hernández et al. (2013) used the intermittent cue-switching task, in which the task cues were divided into implicit and explicit, to test the switching ability of Spanish bilingual and monolingual college students. It turned out that the restart cost of bilinguals was lower in the case of implicit cues. Garbin et al. (2011) performed a non-verbal task on Spanish-Catalan bilinguals and Spanish monolinguals. They found that bilinguals had lower switching costs than monolinguals. Some researchers have used the Wisconsin Card Sorting Task (WCST) to find significant advantages in the switching ability of bilinguals in Chinese-English bilinguals (Dong and Xie, 2014). Also, researchers have suggested the influence of language experience on working memory. Feng (2009) investigated the breadth of digital and spatial location memory in monolinguals and bilinguals. They found that bilinguals had significantly better memory effects in spatial location memory than monolinguals, indicating that the advantage of bilinguals’ memory was reflected in their excellent ability to organize memory materials. Baker (2013) argued that there might be other ways in which bilingual experience could improve working memory, and participants were asked to retest the task 5 days after they had completed the immediate recall test. After 5 days, the results indicated that the target memory of bilinguals was significantly better than that of monolinguals, showing that the bilingual experience slowed down the forgetting process. Bialystok et al. (2014) explored the differences between verbal and non-verbal working memory tasks in monolinguals and bilinguals. They found that bilinguals only had an advantage in non-verbal tasks, and the differences in verbal tasks were insignificant. Although the above studies have amply demonstrated a bilingual cognitive advantage effect, some other researchers have not found that bilinguals have an advantage over monolinguals in executive function (Paap and Greenberg, 2013; Paap et al., 2015), and the results of bilingual advantage effects remain controversial (Antón et al., 2019). Overall, previous studies have mostly paid attention to the experimental contexts under abstract, purely cognitive stimuli and ignored the experimental settings under emotional stimuli.

Emotion is a motivation patterned with perception, cognition, and motor responses. The relationship between emotion and executive function, in a broad sense, is the relationship between emotion and cognition, which has always been the focus of psychologists. Hoffmann put forward the system theory of how emotion and cognition interact. He believed that cognitive processing inevitably involves emotions and positively or negatively impacts cognition. Meanwhile, cognition, in turn, regulates emotion. Emotional processing has an advantage in the early stage of information processing, while in the late stage, such cognitive factors as attentional load and cognitive resources are likely to regulate emotional processing. Accordingly, emotional and cognitive processing can work together interactively to influence the completion of the target task (Bradley, 2009). According to resource limitation theory, individuals may consume limited cognitive resources to suppress irrelevant emotional information when performing complex cognitive tasks (Pessoa, 2009). The relationship between language and emotions is also quite close, and studies of school-age children have found that the development of language skills leads to improved emotional abilities. Pons et al. (2003) noted that children’s language proficiency and age could explain 72% of the variation in emotional perception, with language proficiency independently demonstrating 27%. This evidence implies that emotional perception is not only related to the acquisition of emotional words but also influenced by the level of language development, which indicates that language is an essential factor in constructing emotional perception. The study of emotional words is vital evidence for exploring the relationship between language and emotion. For example, some researchers have found that children’s recognition of emotional words is more accurate and higher in priority than the recognition of emotional faces by using emotional judgment tasks. This means that in early childhood development, the acquisition and application of emotional words precede the acquisition of facial expressions (Michael et al., 2018). Other researchers using story-and emotion-matching tasks have found that emotional words have more direct and accurate cueing effects on early emotional perception (Alonso-Alberca et al., 2012). The story used in the experiment described the cause and process of emotional events. The presentation of emotions was divided into two types: emotional words and emotional face pictures. Children matched the story with the right emotional words or pictures. The results suggested that compared with picture matching, the emotional words selected by the participants were more consistent with the emotions expressed in the story. It shows that in the development of emotional perception, children tend to associate emotional perception with familiar emotional words to obtain accurate emotional information.

Executive function can impact emotions, specifically the ability to regulate emotions (Valian, 2015). Emotion regulation is the ability to accomplish task goals by maintaining, adjusting, inhibiting, or strengthening emotional arousal (Fabes and Eisenberg, 1997). Emotion regulation plays a crucial role in interpersonal communication, social adjustment, academic attainment, and personality development (Eisenberg and Morris, 2003). The frontal, parietal lobe and anterior cingulate cortex (ACC) are brain regions closely related to executive function (Fair et al., 2007), and the prefrontal cortex (PFC) and ACC are also essential brain regions involved in emotion regulation (Phillips et al., 2008). To have control over when and how we experience emotions is thought to be impacted by higher cognitive processes, such as executive functions (Lyusin et al., 2022). Individuals with solid executive function can ensure more effective inhibition, switching and updating of emotional interference stimuli, better regulate attention resources to obtain task-related information, and ignore emotional interference information to optimize task processing and realize more effective allocation and utilization of cognitive resources, thus achieving a more influential role in emotion regulation (Melnick and Hinshaw, 2000; Rößner et al., 2016). If bilinguals have a cognitive advantage on executive function, can bilinguals also be better at regulating their emotions than monolinguals? This has yet to be considered in previous studies. Therefore, whether the bilingual advantage effect in executive function still exists in the more specific and complex context of emotional interference conditions is the focus of this study.

As the implicit mental processing, executive function is not explicitly performed by individuals, and thereby it needs to be studied through traditional behavioral experiments and ERP technology. Due to its high temporal resolution, ERP technology can be effectively used to study the micro-psychological process. In addition, ERP techniques can measure the entire process, from stimulus appearance to behavioral response, and continue to identify the specific stages of cognitive processing affected by the stimulus. The indicators of behavioral experiments are mainly correctness and response time, which reflect the results at the epiphenomenal level and do not effectively provide indicators at the cognitive neuroscience level. Therefore, the ERP technique has a significant advantage in studying executive functions. Previous researchers have conducted a series of research on the neural mechanism of executive function using ERP technology. Some researchers found that such components in ERP as N1, N2, P2, and P3 are closely related to executive function (Petruo et al., 2017). As an ERP component related to early selective visual attention, the N1 component is in the early attention stage of information processing, and its peak value is generally believed to be within 70–100 ms; N1 primarily reflects the allocation of processing capacity for input stimuli and the active filtering of information processing (Hamilton et al., 2014). The P2 component indicates the individual’s rapid perception of the features of stimuli, and the presence of P2 may be related to attention in perceptual processing (Xin et al., 2010). The N2 component is mainly involved in cognitive perception and monitoring, reflecting the allocation of attention resources and the speed of awareness of stimuli, of which the N2 latency period represents the speed of response conflict awareness. The increase in the N2 amplitude indicates that the participant may have invested more cognitive resources in task processing to overcome the effect of distracting stimuli on the current target stimulus (Yuan et al., 2008). The P3 component is related to the allocation of cognitive resources, and its latency mainly reflects the speed of encoding and recognizing stimulus information. At the same time, its amplitude may represent the degree of attention to task-related information. The P3 activity in the central parietal zone is a direct indicator of inhibitory processing control, which can objectively reflect higher cognitive processes such as recognition and judgment in the brain (Bokura et al., 2001).

Overall, then, to explore the influence of bilingual experience on executive function under emotional interference, the present study adopted positive, negative, and neutral emotional stimuli integrated with ERP technology to perform the emotional Simon task, emotional, cognitive switching task, and emotional N-back task in-between the groups of proficient and non-proficient Chinese-English bilinguals. The present study hypothesizes that the bilingual advantage effect may exist under emotional interference.

## 2. Methods

### 2.1. Participants

*A priori* power analysis with G*Power 3.1 (Faul et al., 2007) was conducted to estimate the minimum sample size. Referring to the results of the formal experiment, the experiment could obtain a moderate effect amount. The effect amount (*η*^2^*p* = 0.25, *α* = 0.05, 1-β = 0.95) in repeated measures analysis of variance (ANOVA) was set, and the correlation between the repeated measure levels was low; 0.4 was used as the correlation coefficient. The priori power analysis showed that the total sample size of *N* = 36 was required to observe a significant effect. In this paper, 50 university students from China were recruited for experiments, 45 were finally included with valid data for research, and the sample size was sufficient. Of the 45 participants (M age = 24, 10 men and 35 women), 25 were proficient Chinese-English bilinguals, and 20 were non-proficient Chinese-English bilinguals. Proficient and non-proficient bilinguals were university or graduate students in the same city with the same economic status and similar intellectual levels. All participants were right-handed, with normal or corrected-to-normal vision and standard hearing capabilities; they had no physical disability and no history of mental illness. The inclusion criteria were as follows: proficient bilinguals were those who had passed TEM-8 (Test for English Majors-Band 8, the highest professional English test in China), while the non-proficient were those who had not passed CET-4 (College English Test Band 4, the passing of which equals a score of 5 in International English Language Testing System) yet; Besides, all participants voluntarily participated in the experiment, gave their written informed consent prior to the present study, and received monetary compensation after completing the experiment. This study was approved by the local ethics committee.

### 2.2. Materials

Thirty face pictures, including 10, each representing happy, neutral, and sad faces, with a male-to-female ratio of 1:1, from the Chinese Facial Affective Picture System (CFAPS). Pre-experiments were conducted before the formal experiment, and the experimental pictures were evaluated. The results showed that the faces were well-gendered. Each face image was 5.9 cm × 6.9 cm in size on the screen, with 260 × 300 dpi pixels, randomly rendered on the left or right side of the screen. The screen was located at a distance of 60 cm, directly in front of the participants’ eyes. The stimuli were presented on a CRT monitor with a resolution of 1,024 × 768 and a screen size of 15 inches.

### 2.3. EEG data recording and analysis

The EEG was continuously recorded using 64 electrodes mounted in an electrocap. Horizontal eye movements were measured by deriving the electrooculogram (EOG) from the electrode placed to the lateral of the participants’ right eyes. The reference electrode was set as a reference. Ground points were the midpoint of FPz and Fz. The sampling frequency of all signals was 500 Hz, and the impedance was kept below 50 kΩ. EEG data were continuously recorded, and raw data were offline-analyzed using BP Vision Analyzer Software (Version 2.0). Finally, the same kind of data was superposed after baseline correction and averaging, and the average EEG waveform of each participant after cue presentation was obtained.

## 3. Experiment 1

### 3.1. Hypothesis

Regarding behavioral indicators, the main effect of bilingual experience may be significant. Compared with non-proficient bilinguals, proficient bilinguals will perform better on emotional Simon tasks, reflected in shortened reaction time (RT), improved accuracy, and improved emotional inhibition control. In terms of EEG indicators, in the amplitude and latency of the four main EEG components (N1, N2, P2, and P3), the main effect of bilingual experience may be significant, which can be reflected in the smaller amplitude, shorter latency and better control of emotional inhibition than non-proficient bilinguals.

### 3.2. Procedure

**Figure 1:**
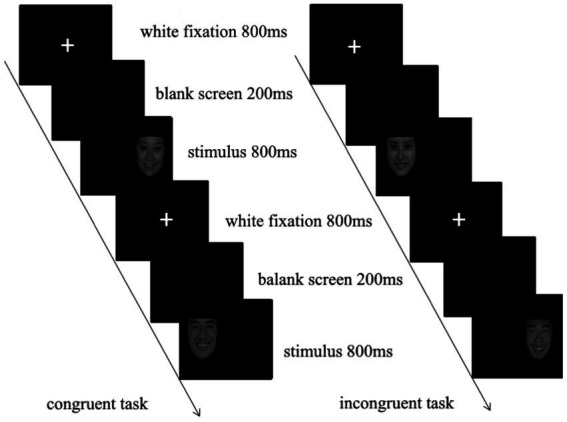
Emotional Simon task.

The classic Simon task was adapted to add three emotional faces as emotional interference stimuli. Participants were tasked with judging the “gender of the face” of the presented face while ignoring its position. They pressed the “Q” key with the index finger of the left hand if the facial expression of the presented face picture was male; if the facial expression was female in the picture, they pressed the “P” key with the index finger of the right hand. There were 383 trials in this experiment, divided into three blocks, each block with 128 trials, and each block contained an equal number of trials for various conditions. Twenty-four trial exercises were provided before the formal experiment, so the participants could clearly understand the experimental tasks and familiarize themselves with the buttons. The specific process is shown in Figure 1.

### 3.3. Results

#### 3.3.1. Behavioral experiment

The Accuracy (ACC) and reaction time (RT) of the inhibitory control task are statistically tested, and the descriptive statistical results are shown in Table 1:

(1) Accuracy

**Table 1:** Descriptive statistical table of behavioral experiment results of inhibitory control tasks for participants with different proficiency.

Emotion type	Task type	Participant type	
		Non-proficient bilinguals (M ± SD)	Proficient bilinguals (M ± SD)
**Positive**	ACC of congruent task	0.93 ± 0.04	0.95 ± 0.04
	ACC of incongruent task	0.92 ± 0.06	0.93 ± 0.07
	RT of congruent task	593.92 ± 43.57	585.91 ± 47.18
	RT of incongruent task	603.29 ± 40.45	604.12 ± 54.89
	Simon effect	9.37	18.21
**Neutral**	ACC of congruent task	0.93 ± 0.04	0.92 ± 0.05
	ACC of incongruent task	0.92 ± 0.07	0.92 ± 0.05
	RT of congruent task	614.26 ± 47.72	591.24 ± 47.08
	RT of incongruent task	617.80 ± 43.15	595.90 ± 46.30
	Simon effect	3.54	4.66
**Negative**	ACC of congruent task	0.91 ± 0.06	0.89 ± 0.06
	ACC of incongruent task	0.88 ± 0.10	0.89 ± 0.08
	RT of congruent task	602.97 ± 41.10	584.60 ± 47.91
	RT of incongruent task	605.65 ± 49.32	601.67 ± 53.79
	Simon effect	2.68	17.07

A 2 (participant type: proficient bilingual vs. non-proficient bilingual) × 3 (emotional type: positive vs. neutral vs. negative) × 2 (task type: congruency vs. incongruency) repeated measures analysis of variance (ANOVA) was conducted on the response accuracy and found that the main effect of the emotion type was significant [*F*(2, 43) = 11.86, *p* < 0.01, partial *η*^2^ = 0.22]; the ACC of the positive emotion group was significantly higher than that of the negative emotion group, and other results were not significant.

(2) Reaction times (RTs)

Repeated measures ANOVA was performed on the RTs of the task. It was found that the main effects of the task type were significant [*F*(1, 44) = 7.05, *p* < 0.05, partial *η*^2^ = 0.14], and the RTs of the congruent task group were significantly shorter than the incongruent task group, and other results were not significant.

(3) Simon effect

The Simon effect is obtained by subtracting between the RTs of incongruent and congruent tasks. Repeated measures ANOVA on the Simon effect revealed that the main effect of the emotion type was significant [*F*(1, 43) = 5.94, *p* < 0.01, partial *η*^2^ = 0.12]; the Simon effect size in the neutral emotion group was significantly smaller than that in the positive emotion group, and the other results were not significant.

#### 3.3.2. ERP

(1) The N1 component

The repeated measures ANOVA of 2 (participant type: proficient bilingual vs. non-proficient bilingual) × 3 (emotion type: positive vs. neutral vs. negative) × 2 (task type: congruency vs. incongruency) was performed, and the results showed that the main effect of the participant type in the N1 amplitude of the frontal region was significant [*F*(1, 44) = 6.93, *p* = 0.01, partial *η*^2^ = 0.14], in which the N1 amplitude (−2.39 μv) of the proficient bilinguals was significantly smaller than that of the non-proficient bilinguals (−4.02 μv). The main effect of the participant type in the N1 amplitude of the central region was significant [*F*(1, 44) =12.29, *p* < 0.01, partial *η*^2^ = 0.23], among which the N1 amplitude (−2.17 μv) of proficient bilinguals was significantly smaller than that of non-proficient bilinguals (−3.62 μv).

(2) The N2 component

**Figure 2:**
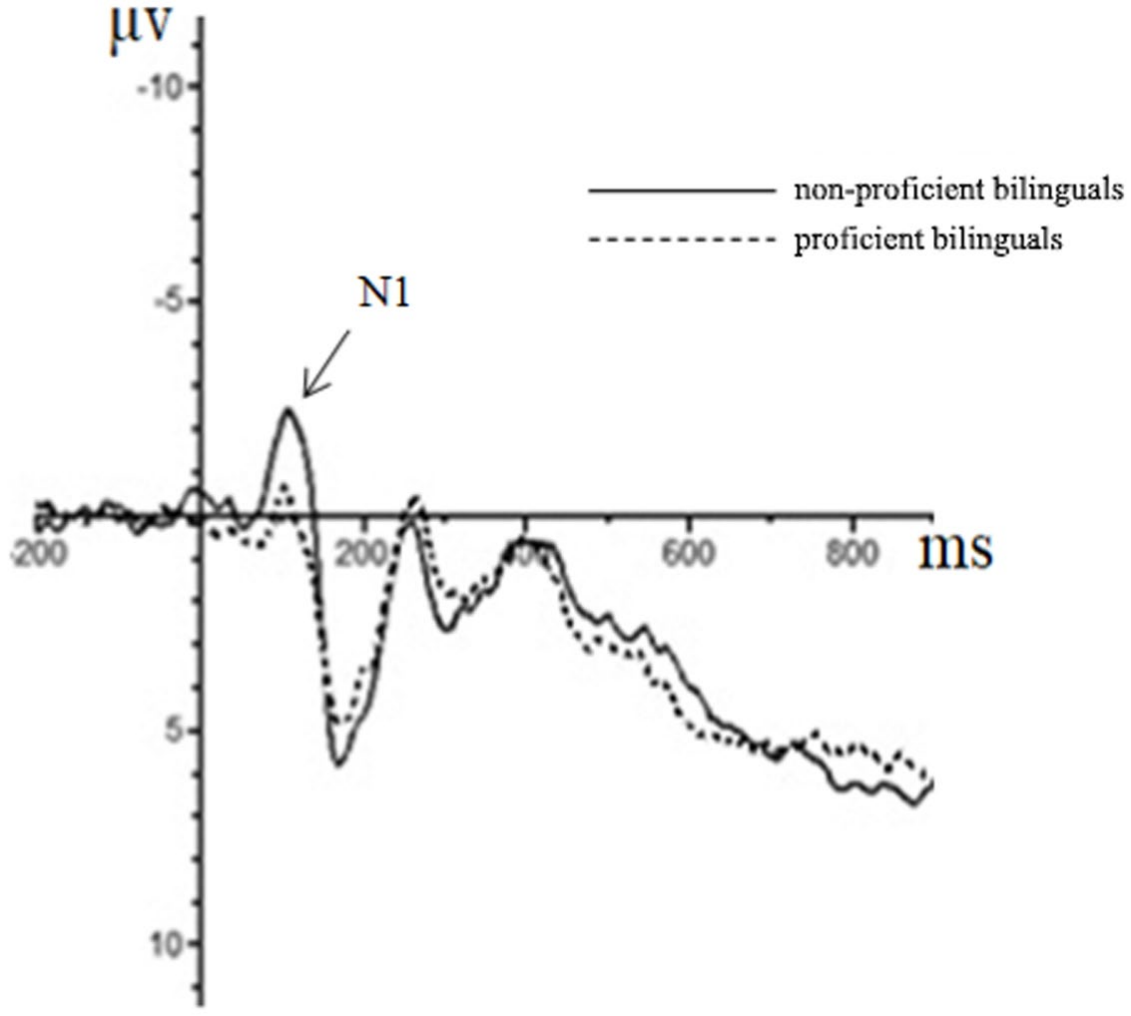
The brainwaves of participant type in Fz electrode.

**Figure 3:**
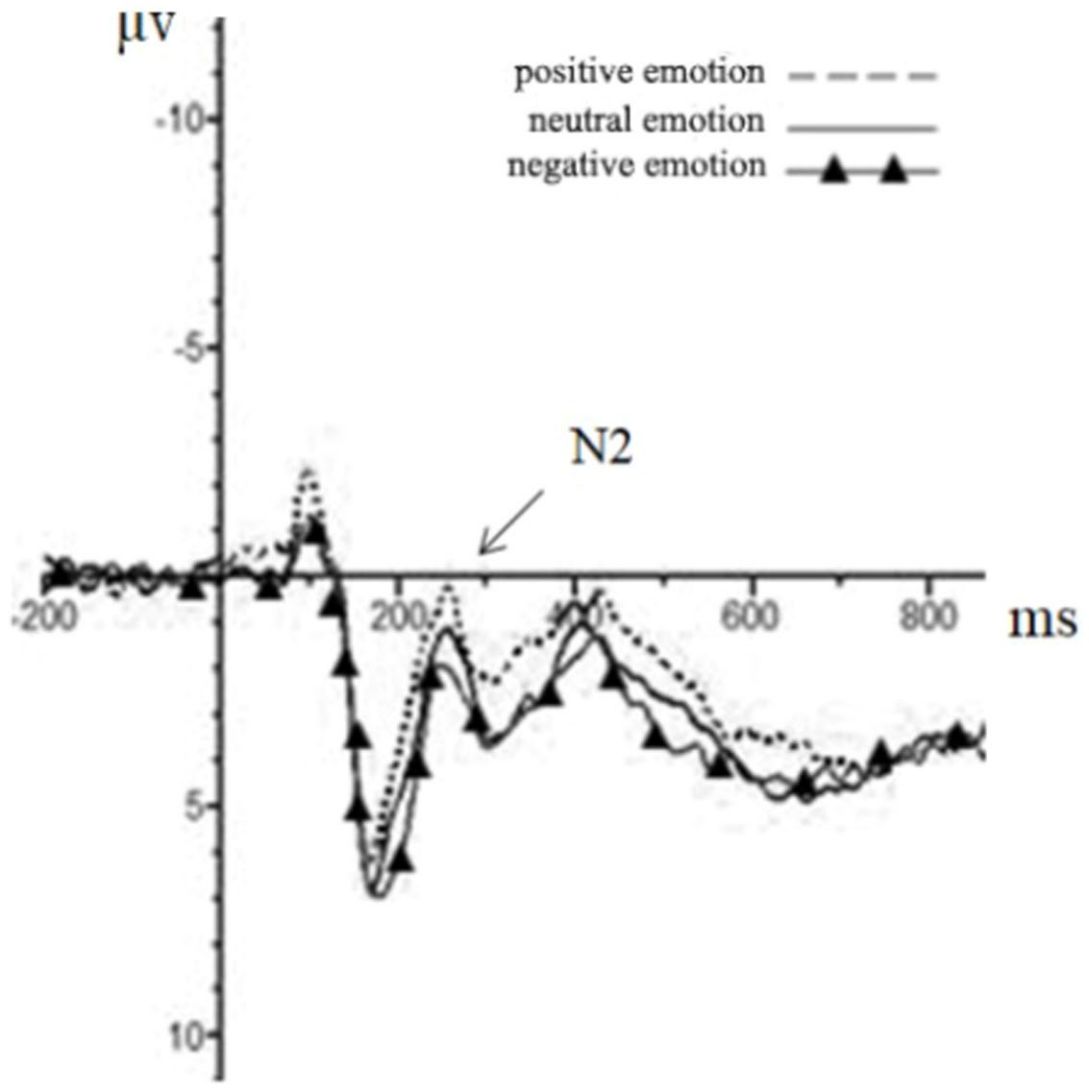
The brainwaves of emotion type in Cz electrode.

**Figure 4:**
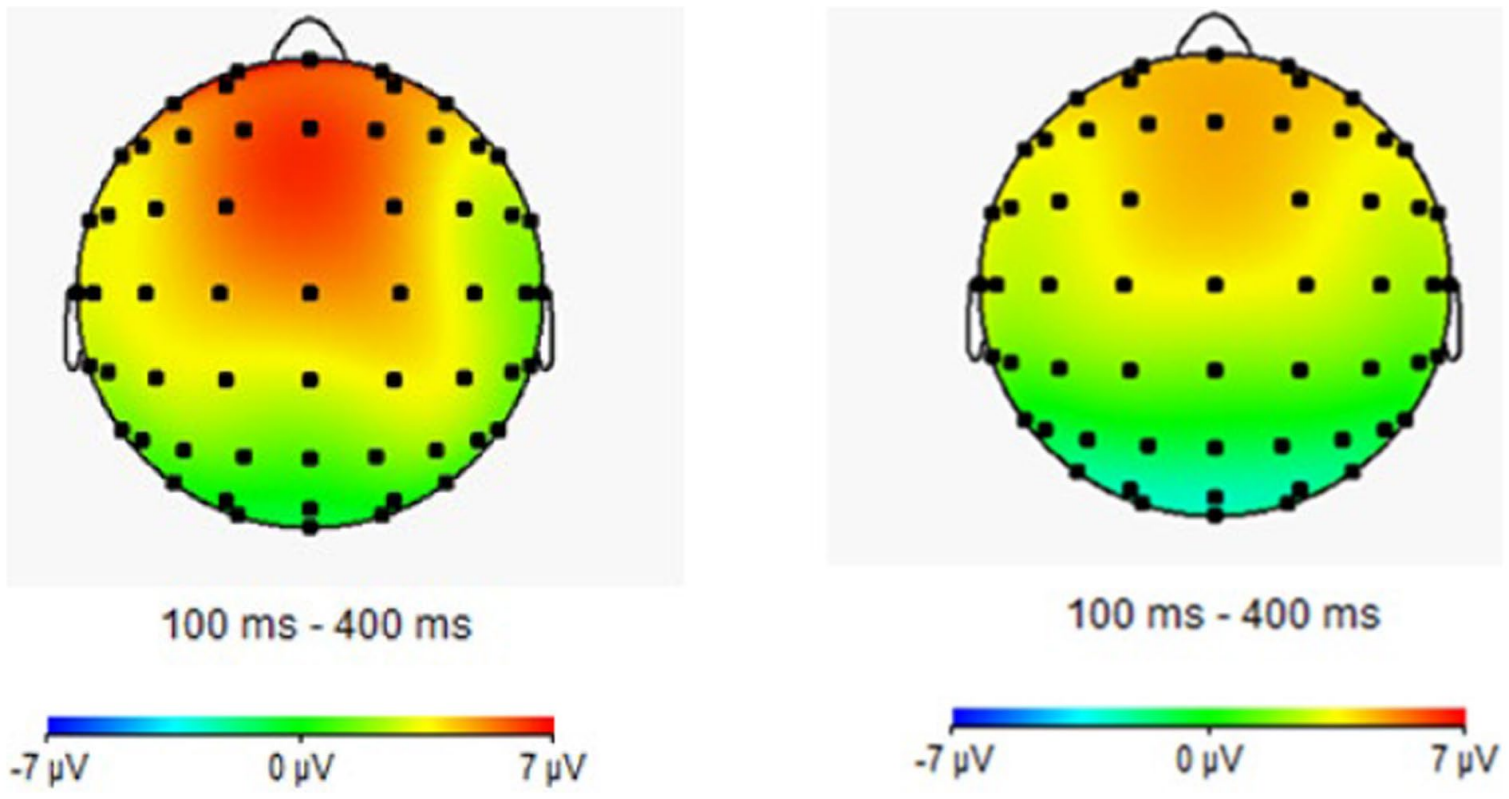
Brain electrical activity mapping (BEAM) of proficient and non-proficient bilinguals.

Repeated measures ANOVA was carried out, and it was found that the main effect of emotion type was also significant in the N2 amplitude of the central region [*F*(1, 44) = 5.37, *p* = 0.01, partial *η*^2^ = 0.11], in which the N2 amplitude (−0.76 μv) of positive emotion was significantly greater than that of neutral emotion (−0.18 μv) and negative emotion (0.39 μv). The main effect of the emotion type in the N2 amplitude of the parietal region was significant [*F*(2, 43) = 10.23, *p* < 0.01, partial *η*^2^ = 0.19], in which the N2 amplitude of positive emotion (−0.45 μv) was significantly more extensive than that of neutral emotion (0.58 μv) and negative emotion (1.46 μv). The results for the remaining ingredients were not significant. The EEG results are shown in Figures 2–4.

### 3.4. Analysis

#### 3.4.1. Behavioral experiment

In inhibitory control tasks, the accuracy decreased, suggesting that negative emotions hindered inhibitory control. Regarding reaction times, the main effect of task type was significant, indicating that the inhibition task could be realized. According to the Simon effect, the main effect of emotion type is significant, and the effect of positive emotion is significantly higher than that of neutral emotion, mainly reflected in the shorter RT of congruent tasks under the condition of positive emotion. The results suggest that positive emotion promotes inhibitory control.

In general, the interference of emotion with inhibitory control is mainly reflected in the hindrance effect of negative emotion and the promotion of positive emotion. The results of the behavioral experiment seem to suggest that the bilingual advantage effect is not significant, which shows that in the process of cognitive processing, the bilingual advantage is difficult to manifest because of emotional interference.

#### 3.4.2. ERP

Although it is difficult to identify the advantage effect of bilingual experience in behavioral indicators, the amplitude of the N1 component had a significant main effect in both the frontal and the central zone. This suggests that bilingual experience has improved task goal awareness in the early attention stage. In addition, the emotional interference effect still existed. As seen from the results of the N2 component, the main effect of the emotion type was significant in the central and the parietal zone, and the amplitude of positive emotions increased, indicating that positive emotions induced a more significant interference effect. In the P3 component, the main effect of the participant type was not significant, which was also consistent with the results of the behavioral experiment. This demonstrated that the emotional interference effect produced by positive emotions masked the influence of language experience in the later task processing, although the bilingual experience in the early attention stage improved the individual’s awareness of the task goal.

## 4. Experiment 2

### 4.1. Hypothesis

Concerning behavioral indicators, the main effect of bilingual experience may be significant. Compared with non-proficient bilinguals, proficient bilinguals have shorter RT to complete emotional and cognitive switching tasks, higher accuracy rates, and better emotional and cognitive conversion ability. In terms of EEG indicators, the amplitude and latency of the four main EEG components (N1, N2, P2, and P3), the main effect of bilingual experience may be significant, and compared with non-proficient bilinguals, proficient bilinguals will have smaller amplitude, shorter latency and better emotional, cognitive switching ability.

### 4.2. Procedure

**Figure 5:**
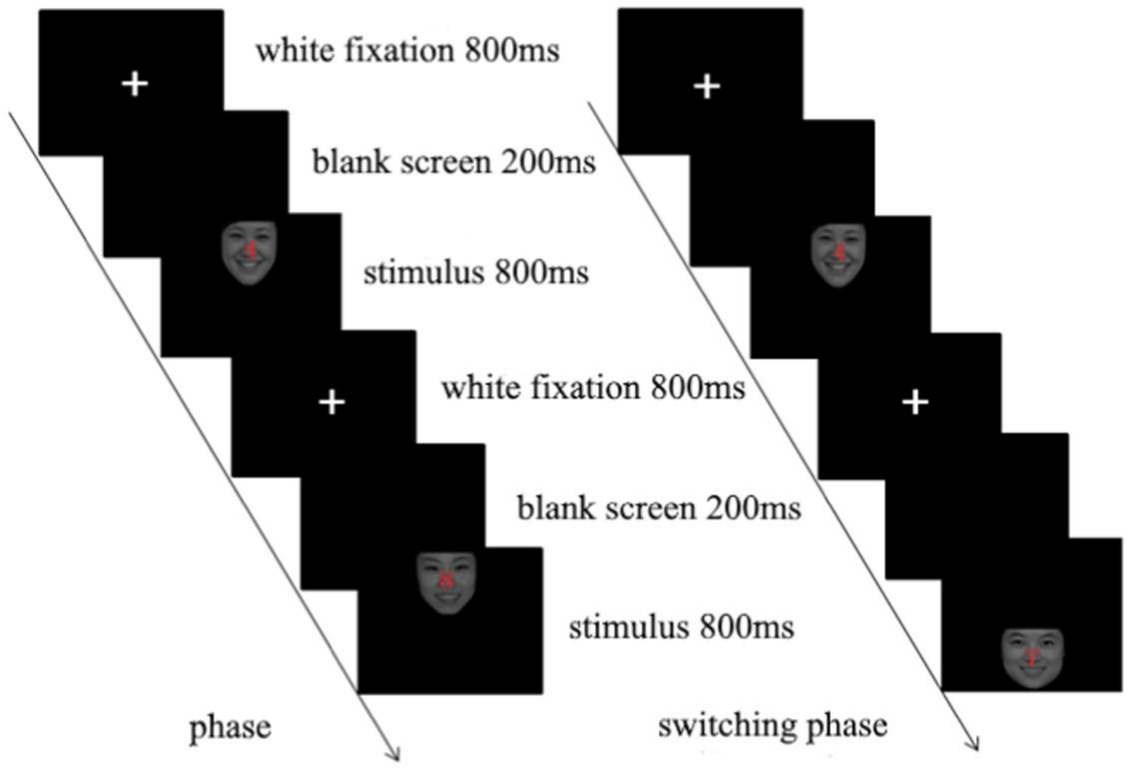
Task of emotional, cognitive switching.

The classic numeric-letter switching task is adapted to add three emotional faces as emotional interference stimuli. The primary task was divided into two parts: when the picture face appeared above the fixation point “+,” the participant judged the numerical size of the presented face, less than or equal to 4 pressed the “Q” key, greater than 4 pressed the “P” key; When the face picture appeared below the fixation point “+,” the participants judged the odd number of the face and pressed the “Q” key, and the even number pressed the “P” key. The participants went through repeating and switching phases when the formal experiments began. The situation that the task request was congruent repeatedly appeared in 32 consecutive trials, followed by a switching phase in which 32 successive trials of the random switching task were conducted. There were 383 trials in this experiment, divided into three blocks, each block with 128 trials, and each block contained an equal number of trials for various conditions. There were 24 trial exercises before the formal experiment, so the participants could clearly understand the experimental tasks and familiarize themselves with the buttons. The specific process is shown in Figure 5.

### 4.3. Results

#### 4.3.1. Behavioral experiment

The Acc and RTs of the switching task are statistically tested, and the descriptive statistical results are shown in Table 2:

(1) Accuracy

**Table 2:** Descriptive statistical table of behavioral experiment results of cognitive switching tasks for participants of different proficiency levels.

Emotion type	Task type	Participant type	
		Non-proficient bilinguals (M ± SD)	Proficient bilinguals (M ± SD)
**Positive**	ACC of repeating task	0.88 ± 0.04	0.86 ± 0.06
	ACC of switching task	0.80 ± 0.09	0.77 ± 0.11
	RT of repeating task	597.54 ± 50.35	600.53 ± 57.46
	RT of switching task	665.80 ± 88.52	640.24 ± 81.24
	Switching cost	68.26	39.71
**Neutral**	ACC of repeating task	0.92 ± 0.03	0.89 ± 0.08
	ACC of switching task	0.86 ± 0.09	0.82 ± 0.12
	RT of repeating task	572.45 ± 54.29	581.48 ± 62.53
	RT of switching task	639.42 ± 72.67	627.57 ± 78.53
	Switching cost	66.97	46.09
**Negative**	ACC of repeating task	0.93 ± 0.04	0.90 ± 0.07
	ACC of switching task	0.84 ± 0.08	0.81 ± 0.12
	RT of repeating task	569.70 ± 77.63	578.21 ± 68.85
	RT of switching task	626.96 ± 86.68	626.53 ± 91.91
	Switching cost	57.26	48.32

A 2 (participant type: proficient bilingual vs. non-proficient bilinguals) × 3 (emotion type: positive vs. neutral vs. negative) × 2 (task type: repeating vs. switching) repeated measures ANOVA was conducted to examine the accuracy rate of response, and the results showed that the main effect of emotion type was significant [*F*(2, 43) = 22.47, *p* < 0.01, partial *η*^2^ = 0.35], and the accuracy of positive emotion group were significantly lower than that of negative emotion group. The main effect of the task type was significant [*F*(1, 44) = 55.63, *p* < 0.01, partial *η*^2^ = 0.58], in which the accuracy of repetition task groups was significantly higher than that of switching task groups, and other results were not significant.

(2) RTs

Repeated measures ANOVA was carried out on the RTs of the task, and it was found that the main effect of the emotion type was significant, [*F*(2, 43) = 13.36, *p* < 0.01, partial *η*^2^ = 0.24], and the positive emotion group had a significantly longer response than the negative emotion group. The main effect of task type was significant [*F*(1, 44) = 102.04, *p* < 0.01, partial *η*^2^ = 0.71]; the RTs of switching task groups were significantly longer than that of repeating task groups; other results were not significant.

(3) Switching cost

By subtracting the RT of the switching task and the repetition task, the switching cost is shown in Table 2. A 2 (participant type: proficient bilingual vs. non-proficient bilinguals) × 3 (emotion type: positive vs. neutral vs. negative) repeated measures ANOVA was conducted for switching cost, and the results showed that the main effect of emotion type was not significant [*F*(2, 43) = 0.10, *p* = 0.90]; the main effect of the between-group was not significant [*F*(1, 44) = 3.26, *p* = 0.08].

#### 4.3.2. ERP

(1) The N1 component

A 2 (participant type: proficient bilingual vs. non-proficient bilingual) × 3 (emotion type: positive vs. neutral vs. negative) × 2 (task type: vs. switching) repeated measures ANOVA was performed. The results showed that in the N1 amplitude of the central zone, the main effect of the participant type was significant [*F*(1, 44) = 4.77, *p* = 0.04, partial *η*^2^ = 0.10], of which the N1 amplitude (−4.97 μv) of non-proficient bilinguals was significantly greater than that of proficient bilinguals (−3.08 μv).

(2) The N2 component

Repeated measures ANOVA was carried out, and it was found that the main effect of the task type was significant in the N2 amplitude of the frontal zone [*F*(1, 44) = 8.06, *p* < 0.01, partial *η*^2^ = 0.16], in which the N2 amplitude (0.25 μv) of the repeating type was significantly smaller than that of the switching type (−0.57 μv). In the N2 amplitude of the central zone, the main effect of the task type was significant [*F*(1, 44) = 21.78, *p* < 0.01, partial *η*^2^ = 0.34], in which the N2 amplitude (0.51 μv) of the repeating type was significantly smaller than that of the switching type (−0.78 μv). The main effect of the task type was significant in the N2 amplitude of the parietal zone [*F*(1, 44) = 26.72, *p* < 0.01, partial *η*^2^ = 0.38], in which the N2 amplitude of the repeating type (0.51 μv) was significantly smaller than that of the switching type (−0.70 μv).

(3) The P2 component.

Repeated measures ANOVA showed that the main effect of the task type in the P2 amplitude of the central zone was significant [*F*(1, 44) = 12.43, *p* < 0.01, partial *η*^2^ = 0.22], in which the P2 amplitude of the repeating type (13.78 μv) was significantly greater than that of the switching type (12.64 μv). The main effect of the task type was significant in the P2 amplitude of the parietal zone [*F*(1, 44) = 43.00, *p* < 0.01, partial *η*^2^ = 0.34], in which the P2 amplitude of the repetition type (8.50 μv) was significantly more extensive than that of the switching type (7.52 μv).

(4) The P3 component.

**Figure 6:**
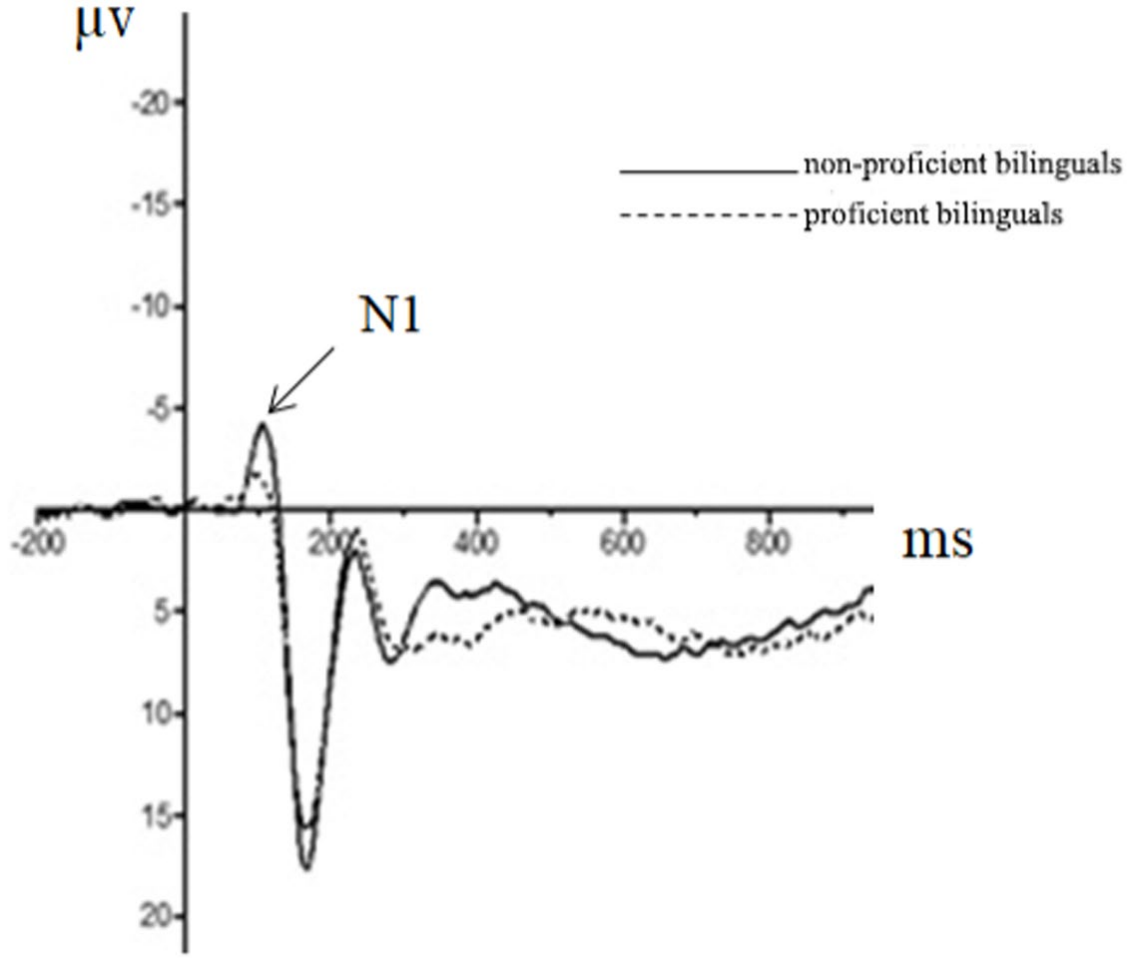
The brainwaves of participant type in Fz electrode.

**Figure 7:**
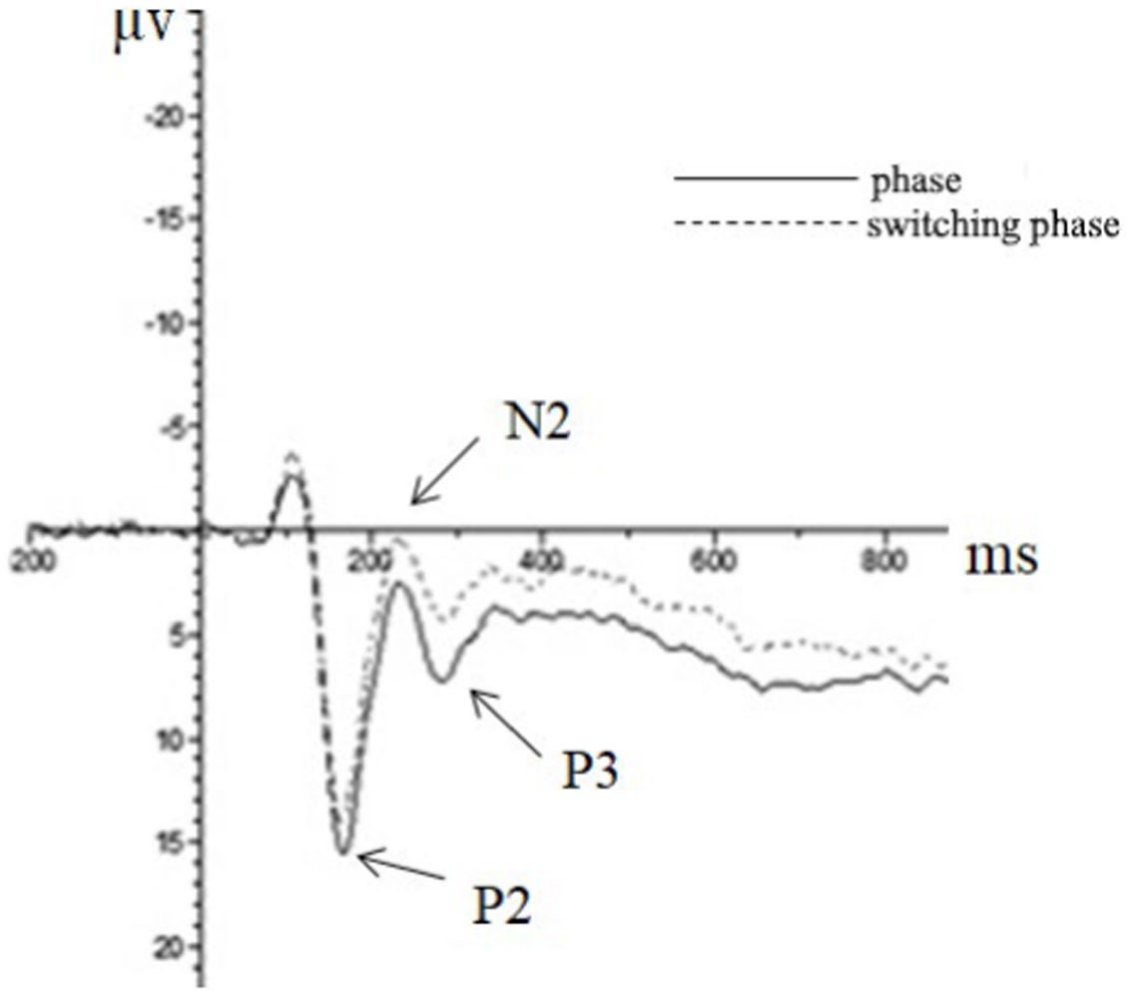
The brainwaves of task type in Cz electrode.

**Figure 8:**
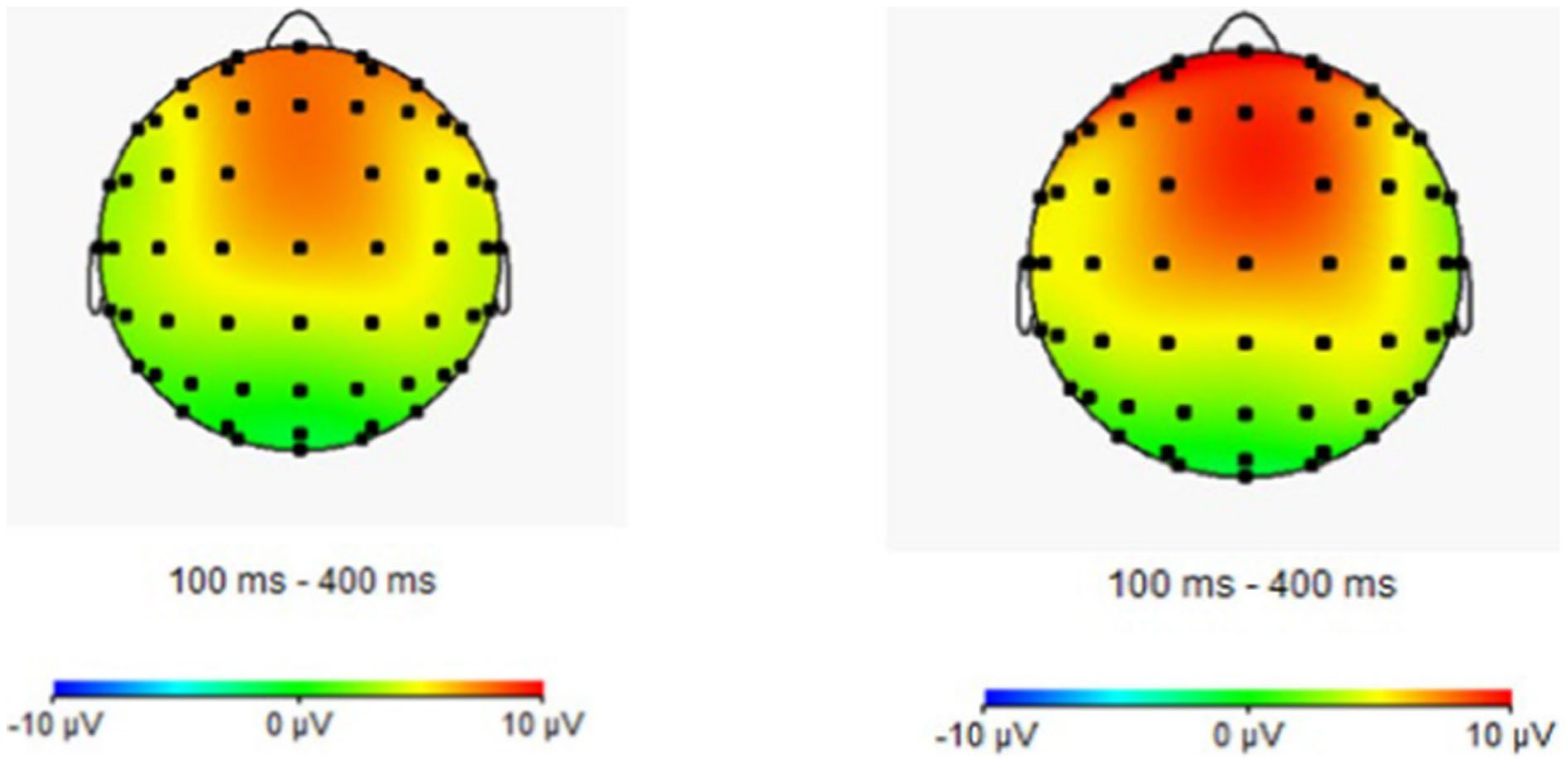
Brain electrical activity mapping (BEAM) of proficient and non-proficient bilinguals.

Repeated measures ANOVA showed that the main effect of the task type was significant in the P3 amplitude of the central zone [*F*(1, 44) = 8.88, *p* < 0.01, partial *η*^2^ = 0.17], in which the amplitude of P3 of the repeating type (9.32 μv) was significantly greater than that of the switching type (8.36 μv), and other results were not significant. The ERP is presented in Figures 6–8.

### 4.4. Analysis

#### 4.4.1. Behavioral experiment

In the switching task, positive emotion hindered the task and showed a decrease in accuracy. The response results showed that the task type’s main effect was significant, indicating that the switching task was realized. The main effect of the emotion type was significant, and the response of positive emotions increased significantly, which suggests that positive emotions play a significant role in interfering with the switching task. In the switching costs, the participant type’s main effect was insignificant, indicating that the bilingual experience was still interfered with by emotions in the switching task.

#### 4.4.2. ERP

Similar to the inhibitory control task, the EEG indicators showed that the main effect of N1 amplitude in the central zone was significant for the participant type, demonstrating that the bilingual experience improved target detection ability in the early attention stage. Correspondingly, the task also induced more obvious N2, P2, and P3 components, and the amplitude of the three components significantly affected the task type. This activation was more evident in the parietal and the central zone, indicating that the switching task required a lot of cognitive resources compared with repeating tasks. The main effect of task switching in emotions was insignificant, which may be because the difficulty in tasks increased the participants’ priority to task switching, thereby ignoring the interference of background emotional stimuli and reducing the interference effect of emotions.

## 5. Experiment 3

### 5.1. Hypothesis

Regarding behavioral indicators, the main effect of bilingual experience may be significant. Compared with non-proficient bilinguals, proficient bilinguals can have shorter reaction time (RT), higher accuracy rate and better emotional working memory in the emotional N-back task. In terms of EEG indicators, the main effect of bilingual experience may be significant in the amplitude and latency of four main components of EEG (N 1, N 2, P 2, P 3). Compared with non-proficient bilinguals, proficient bilinguals can have lower amplitude, shorter latency, and better ability to update emotional working memory.

### 5.2. Procedure

**Figure 9:**
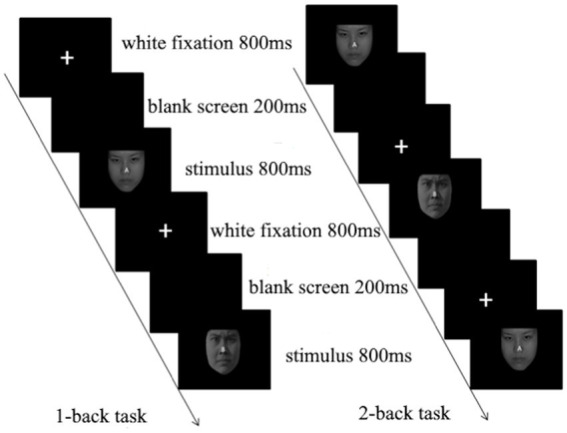
Emotional N-back task.

The classic N-back task was adapted to add three emotional faces as emotional interference stimuli. The primary task was divided into two parts: in the 1-back task, participants were asked to judge whether the letters in the center of the currently appearing image were the same as the letters in the previously presented image, press the “Q” key if they were the same, and press the “P” key if they were different. In the 2-back task, participants were asked to judge whether the letters in the center of the currently appearing image were the same as the letters in the previous image presented, press the “Q” key if they were the same and press the “P” key if they were different. The experiment started with a 1-back task followed by a 2-back task; each task had three blocks, each block had a total of 60 trials, and each block contained an equal number of trials of various conditions. Before the formal experiment, there were 24 trial exercises to enable participants to clearly understand the experimental tasks and familiarize themselves with the buttons. The specific process is presented in Figure 9.

### 5.3. Results

#### 5.3.1. Behavioral experiment

The ACC and RTs of the updating task are statistically tested, and the descriptive statistical results are shown in Table 3:

(1) Accuracy

**Table 3:** Descriptive statistical table of N-back task behavioral experiment for participants with different proficiency.

Emotion type	Task type	Participant type	
		Non-proficient bilinguals (M ± SD)	Proficient bilinguals (M ± SD)
**Positive**	ACC of 1-back task	0.92 ± 0.04	0.93 ± 0.08
	ACC of 2-back task	0.90 ± 0.08	0.91 ± 0.08
	RT of 1-back task	640.18 ± 83.34	649.48 ± 106.38
	RT of 2-back task	734.37 ± 109.68	712.55 ± 112.92
	ACC of 1-back task	0.94 ± 0.05	0.93 ± 0.05
**Neutral**	ACC of 2-back task	0.93 ± 0.05	0.93 ± 0.06
	RT of 1-back task	601.02 ± 64.13	624.66 ± 105.74
	RT of 2-back task	708.33 ± 73.27	691.00 ± 137.90
	ACC of 1-back task	0.93 ± 0.06	0.93 ± 0.06
	ACC of 2-back task	0.93 ± 0.06	0.92 ± 0.08
**Negative**	RT of 1-back task	596.90 ± 74.70	616.21 ± 99.70
	RT of 2-back task	689.51 ± 96.60	680.05 ± 148.05
	ACC of 1-back task	0.92 ± 0.04	0.93 ± 0.08
	ACC of 2-backtask	0.90 ± 0.08	0.91 ± 0.08
	RT of 1-back task	640.18 ± 83.34	649.48 ± 106.38

The repeated measures ANOVA, which 2 (participant type: proficient bilingual vs. non-proficient bilingual) × 3 (emotion type: positive vs. neutral vs. negative) × 2 (task type: 1-back vs. 2-back), was conducted to examine the accuracy of response and found that there was no significant difference in the accuracy of each group.

(2) RTs

Repeated measures ANOVA was performed on task RTs, and it was found that the main effect of emotion type was significant [*F*(2, 43) = 10.39, *p* < 0.01, partial *η*^2^ = 0.24], and the positive emotion group responded significantly longer than the negative emotion task group. The main effects of the task type were significant [*F*(1, 44) = 28.00, *p* < 0.01, partial *η*^2^ = 0.50], and the RTs of the 2-back task group were significantly longer than that of the 1-back task group, and other results were not significant.

#### 5.3.2. ERP

(1) N1 component

A 2 (participant type: proficient bilingual vs. non-proficient bilingual) × 3 (emotion type: positive vs. neutral vs. negative) × 2 (task type: 1-back vs. 2-back) repeated measures ANOVA was conducted, the results showed that the main effect of the participant type was significant in the frontal N1 amplitude [*F*(1, 44) = 5.07, *p* = 0.03, partial *η*^2^ = 0.12], in which the N1 amplitude (−1.69 μv) of the proficient bilinguals was significantly smaller than that of the non-proficient bilinguals (−2.89 μv).

(2) N2 component

Repeated measures ANOVA showed that in the N2 amplitude of the frontal zone, the main effect of the task type was significant [*F*(1, 44) = 4.63, *p* = 0.04, partial *η*^2^ = 0.11], in which the N2 amplitude (−3.31 μv) of the 1-back task was significantly greater than that of the 2-back task (−2.66 μv). In the N2 amplitude of the central zone, the main effect of the task type was significant, [*F*(1, 44) = 6.89, *p* = 0.01, partial *η*^2^ = 0.15], indicating that the N2 amplitude (−3.32 μv) of the 1-back task was significantly greater than that of the 2-back task (−2.54 μv).

(3) P2 component

Repeated measures ANOVA showed that in the P2 amplitude of the central zone, the main effect of the task type was significant [*F*(1, 44) = 4.15, *p* = 0.05, partial *η*^2^ = 0.10], suggesting that the P2 amplitude (1.90 μv) of the 1-back task was significantly smaller than that of the 2-back task (2.50 μv).

(4) P3 component

**Figure 10:**
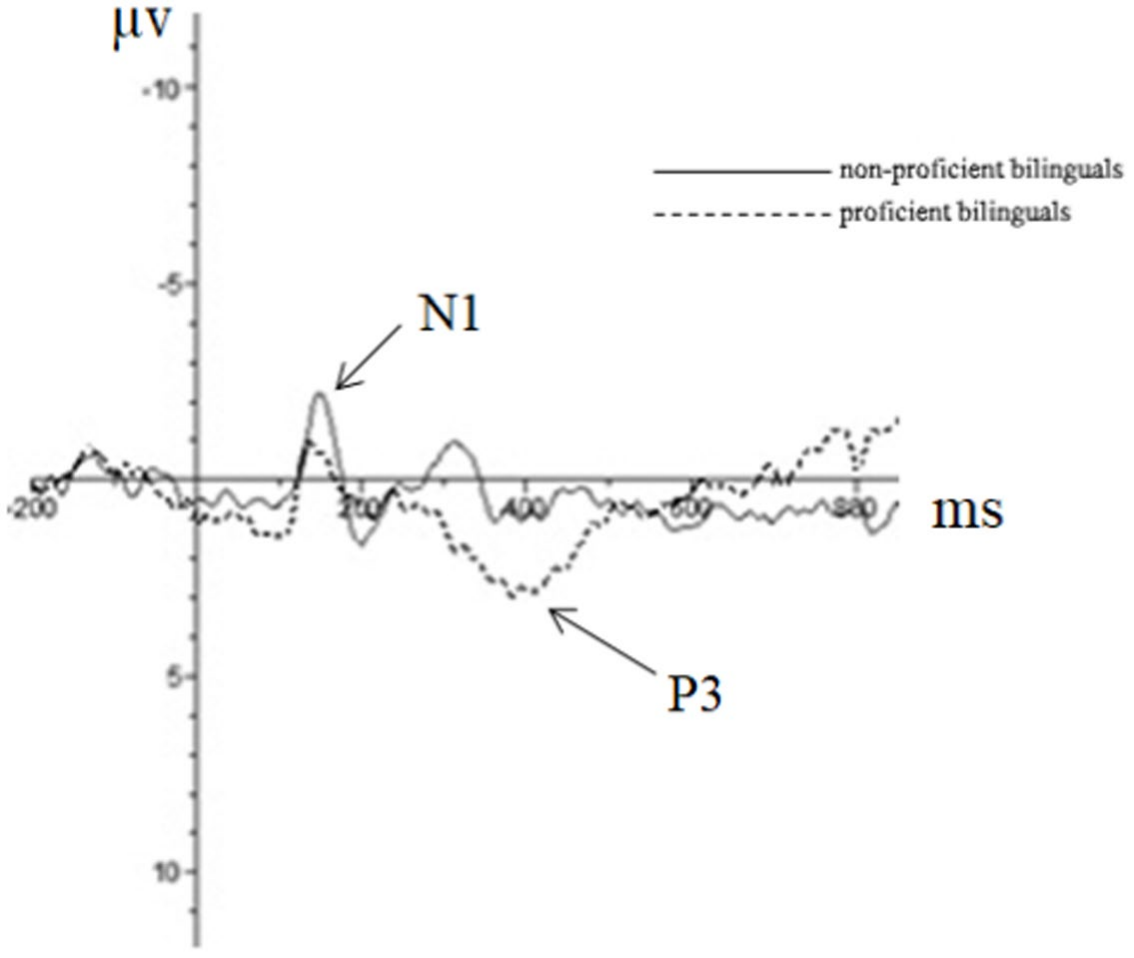
The brainwaves of participant type in Fz electrode.

**Figure 11:**
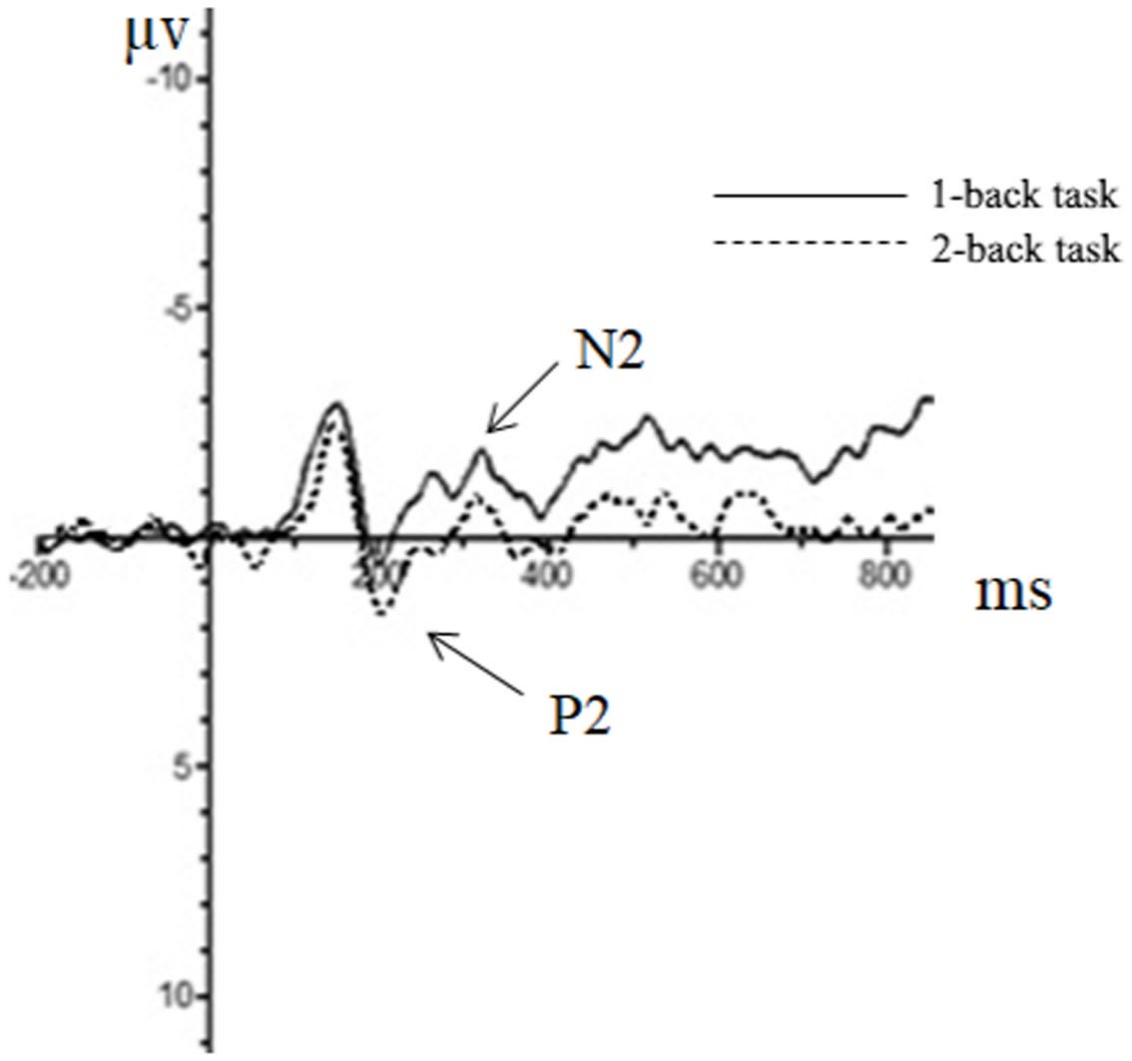
The brainwaves of emotion type in Cz electrode.

**Figure 12:**
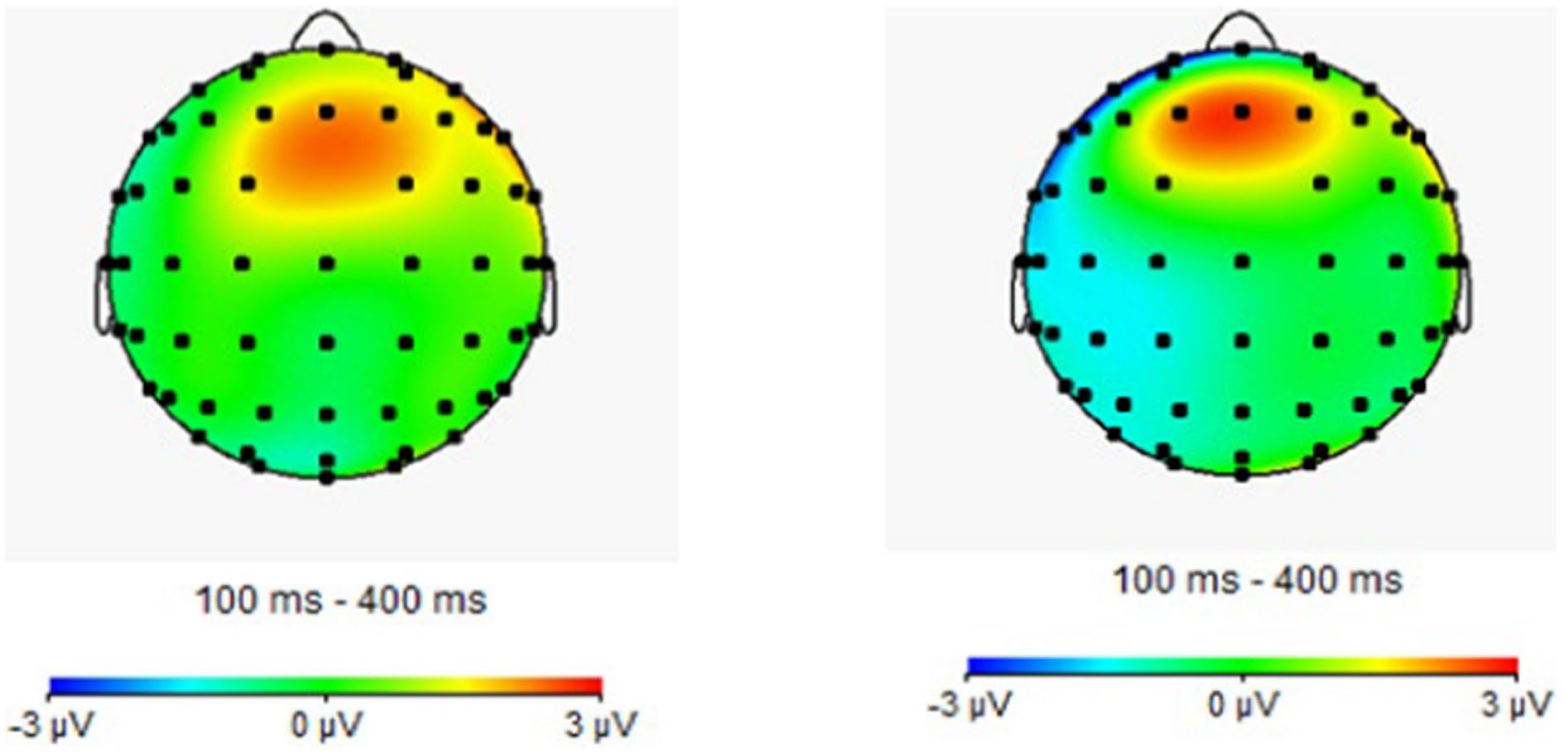
Brain electrical activity mapping (BEAM) of proficient and non-proficient bilinguals.

Repeated measures ANOVA showed that in the P3 amplitude of the frontal zone, the main effect of the participant type was significant [*F*(1, 44) = 5.00, *p* = 0.03, partial *η*^2^ = 0.11], among which the P3 amplitude (2.96 μv) of non-proficient bilinguals was significantly smaller than that of proficient bilinguals (5.04 μv), and other results were not significant. The ERP is presented in Figures 10–12.

### 5.4. Analysis

#### 5.4.1. Behavioral experiment

In the updating task, the main effect of the task type was significant in terms of the RTs, indicating that cognitive load had an impact on the updating of working memory, and a higher load required more cognitive resources than a lower one, thereby prolonging the RTs. The main effect of emotional type was significant, and the RTs of positive emotions increased significantly, indicating a significant interfering effect on the updating task. The bilingual cognitive advantage effect still needs to demonstrate.

#### 5.4.2. ERP

In the three subfunctions of executive function, bilingual experience significantly affected early attention processing. In addition, bilingual experience also affected the late processing of updating. Similar to the results of the previous two experiments, the main effect of the participant type of N1 amplitude was significant in ERP indicators. That is, in the P3 component, the main effect of the participant type was significant. At the same time, the task type was significant in N2 and P2 components, suggesting that the cognitive load influenced the updating function, and the high-load task type required more cognitive resources.

## 6. General discussion

### 6.1. The influence of bilingual experience on executive function under emotional interference

Emotional stimuli are ubiquitous in everyday life. As mentioned above, previous studies on the effect of bilingual experience on executive function are still controversial, and the specific conditions and contexts under which the effect of the advantage effect of bilingual cognition are still uncertain. The present study aims to explore the effects of bilingual experience on executive function and to further investigate its specific neural mechanisms under the interference of three kinds of emotional stimuli.

Based on the hypothesis that bilingual learning can improve students’ academic performance by promoting their executive function, common teaching activities inevitably involve regulating emotional stimuli. Therefore, studying the influence of bilingual experience under emotional interference is vital. In this study, three emotional tasks, namely, the emotional Simon paradigm, the emotional switching ability paradigm, and the emotional N-back paradigm, were used for the experiments. Their fundamental processing mechanisms were explored in conjunction with ERP techniques. The results showed that under emotional interference, the effect of bilingual experience on executive function only existed in the early stage of attention. Proficient bilinguals could detect the stimuli and identify the task targets more quickly. However, emotional stimuli require many cognitive resources in the later stage, thus concealing the influence of language experience.

Moreover, it is noteworthy that differentiating the cognitive activities of proficient and non-proficient bilinguals in different stages of task processing is the main finding and innovation of the present study. First, the ERP results suggested that bilingual experience significantly affected the three subcomponents of executive function, inhibitory control, cognitive switching, and working memory updating in the early N1 component. The amplitude of the N1 component in proficient bilinguals was significantly lower than that in non-proficient bilinguals. In addition, as an early component, the N1 component showed selective attention to information (Yang et al., 2016). In the present study, emotional stimuli were included in the experimental task, and N1 also showed the priming of emotional processing. Therefore, the bilingual experience could still show an advantageous effect on early attention selection and the priming of emotion processing under emotional interference, which suggested that proficient bilinguals could notice emotional stimuli more quickly and distinguish emotional information better.

Previous studies have also found that processing emotional stimuli takes precedence in the brain. This mechanism enables individuals to respond quickly and accurately to emotional stimuli, which shows how organisms have evolved to adapt to their environment. This processing advantage is mainly found in the allocation of attention when emotional materials are presented under the threshold; human beings, as subjects, may be physiologically in response to the corresponding stress (Dolan, 2002). The present study found that in the later period of the experiment, the P3 component was the primary indicator of cognitive resource consumption. There was no significant difference between the two groups, which indicated that bilingual experience, with a particular trend of advantages, could not show its overall cognitive advantages due to the interference of emotional stimuli, which results in the depletion of a large number of cognitive resources of emotions.

The experimental results seemingly show no significant “bilingual cognitive advantage effect” in the late stage of final task processing, which is partly contrary to the hypothesis of this study. The reasons may be: first, although the previous studies have obtained evidence of the “bilingual cognitive advantage effect” in executive function, the scope and existence conditions of this advantage effect are still unclear, and there are many influencing factors in the language itself, such as language experience proficiency, language experience acquisition mode and acquisition environment, acquisition age and other factors, including such individual social factors as socioeconomic status, educational attainment etc. (Morton and Harper, 2007). In this study, the selected group of participants are all college students. It is possible that some proficient bilinguals, affected by their economic conditions and language acquisition environment, have only improved their language proficiency but fail to produce certain cognitive advantages in executive functions. Secondly, task paradigm and task difficulty also restrict language experience’s influence on executive function. Many studies have found no significant difference in performance between bilinguals and monolinguals on simple cognitive tasks and that bilinguals show cognitive advantage only when the task is more complicated. Bialystok et al. (2005) found that bilinguals outperformed monolinguals only when the task required more monitoring and switching. Similarly, Costa et al. (2009) suggested that bilinguals did not show an advantage in all laterally inhibitory tasks but only in those that required a high level of monitoring ability. This indicates that bilingual advantage may be limited to specific domains or in some cognitive functions. Thirdly, the existence of the “bilingual cognitive advantage effect” itself is still controversial. Many researchers have pointed out that acquiring two languages can confuse bilinguals, making the final state of acquisition worse than that of monolinguals. On the one hand, bilinguals can have a distinct disadvantage regarding vocabulary extraction. For example, in the picture naming task, the bilinguals had a longer RT and higher error rate than the monolinguals. On the other hand, there was no significant difference between monolinguals and bilinguals in the stop-signal and saccade tasks (Morales et al., 2013). In cognitive switching, no bilingual cognitive advantage effect was found (Paap et al., 2017). Finally, there is evidence that there is no significant difference between monolinguals and bilinguals in working memory. For example, Ratiu and Azuma (2015) still found no effect of bilingualism on cognitive advantage in tests of working memory capacity in 52 bilinguals and 53 monolinguals. In addition, there is also evidence that language experience does not have a significant advantage effect on the updating function. For example, the study of 9-year-old monolinguals and bilinguals on the emotional N-back task showed that there was still no significant difference in task performance between the two groups (Janus and Bialystok, 2018). The bilingual cognitive advantage might not appear in some specific variables, environments, and different participant groups, suggesting that further experimental verification is still needed.

### 6.2. The influence of emotional interference on the effect of bilingualism

Behavioral experiments suggested that positive emotion and negative emotion had slightly different effects on inhibition, switching, and updating, among which positive emotions promoted inhibition control, while in the task of switching and updating, positive emotions increased the RT of the participants to complete the task, and hinder task processing. In ERP results, the effect of positive emotion on inhibition control was mainly reflected in the N2 component, which induced a more negative N2 amplitude. The possible reason is that positive emotional stimuli induced higher emotional arousal when they appeared. Thus, the speed of response in cognitive monitoring was improved. At the same time, due to the priority of processing, it would induce higher amplitude and occupy more cognitive resources than neutral stimulation. The theory of the dual competition model believes that when emotional stimuli act as the interference information for cognitive conflict, emotional stimuli are easier to be processed because of their processing advantage so they can have a more substantial interference effect on goal-related tasks than neutral distraction stimuli. On top of that, capacity theory believes that processing emotion-related information will promote the activation of brain-wide neural networks. As a result, positive and negative emotional states may receive a higher priority of attention.

Additionally, there were significant emotional interference effects in inhibition control, cognitive switching, or working memory updating, but the specific effects of different emotional valences varied in different tasks. Specifically, in the inhibition control task, because the task judgment involved is mainly about attentional control, positive emotions can improve an individual’s attention awareness, thereby increasing the processing speed (Liu et al., 2009). In addition, the experimental results also found differences in positive emotions’ effects on inhibitory control, cognitive switching and working memory updating. The inhibitory control task took the emotional face as a direct emotional stimulus in the experiment. When the participants identified the gender of the picture, the positive emotions were implicitly processed by the participants, thus producing a positive impact. However, in the cognitive switching and working memory updating tasks, the emotional faces were presented as the emotional background, and the participants needed to perform cognitive tasks even though the emotional background could considerably interfere with cognitive activities. Therefore, due to their higher degree of emotional arousal, positive emotions make the participants consume more cognitive resources, thus creating a hindering effect. In the tasks of switching and updating, the accurate judgment of the task rules and the completion of the memory task required participants to consume a lot of cognitive resources due to the great difficulty and long duration of the task. Although positive emotion could improve the rate of attention and enhance the early attention response, in the later cognitive processing stage, positive emotion would hinder the completion of the task by occupying more cognitive resources and ultimately extending the time of processing the task (Tong et al., 2018). The results of negative emotions in the experiment were seemingly weaker than positive ones. However, there was an entirely different interference trend from neutral emotions, indicating that emotional stimuli interfere with executive function regardless of positive and negative emotions.

This study highlights restoring a common real-life phenomenon: how the emotional interference condition in task processing performs. It explores whether the bilingual advantage effect still exists under this particular condition. Therefore, although emotional stimulus acts as a condition of interference, the difference in the impact of emotional stimuli is not the focus of this study. We did find that emotions affected executive function and interfered with the bilingual advantage effect. In specific interference effects, the positive emotional interference showed a better effect due to its promotion in an individual’s awakening state. In future research, positive emotion can be used as a single emotional stimulus to shorten the experimental time and optimize efficiency.

### 6.3. Limitations and prospects

The experimental results can provide particular references and value for subsequent research. However, the study still has certain limitations in the scope of participants’ selection and the difficulty of the experimental tasks. As suggested, the ability to perform an executive function is significantly different in young children and the elderly. The researchers generally believe that the critical period of executive function is 3 ~ 6 years old, and there are significant differences in children’s cognitive control ability development. The current research results have generally found that the impact of bilingual experience on children’s executive function is significantly different. The studies of different operation paradigms showed that children bilinguals develop a significantly earlier ability of cognitive theory and ability to solve false belief problems than children monolinguals. Their task performance level is considerably better than children monolinguals of the same age (Li et al., 2016). With the increase of age, cognitive ability gradually deteriorates, and executive function decreases, and the elderly with bilingual learning experience have an advantage in the return inhibition of executive function (Jiao et al., 2017). On the other hand, because the task itself increases emotional stimuli and requires a certain number of cognitive resources consumed by the participants, it might not be easy to pursue the speed of reaction to obtain accuracy, which makes the actual completion of the task between proficient bilinguals and non-proficient bilinguals not much different.

Future research should gradually expand the research population, focusing on young children and the elderly, and the influence of additional variables should be excluded as far as possible. The groups with similar socioeconomic status and education levels should be fully considered. What’s more, in the choice of experimental tasks, selecting the experimental paradigm with a certain degree of task difficulty is suggested to achieve better performance of language experience advantage. Finally, fMRI technology is known to have the advantage of more accurate localization of brain regions. Therefore, fMRI technology can be considered for more in-depth research in the future to clarify the basis of brain function in the interaction and the influence of language, emotion and cognition.

## 7. Conclusion

This study explored the effects of bilingual experience on the three subfunctions of executive function: inhibition, switching, and updating under emotional interference. Besides, in combination with ERP technology, the experimental results of ethology and electrophysiology were obtained through the emotional Simon task, the emotional cognitive switching task, and the emotional N-back task. Consequently, based on the comprehensive analysis of the above results, the conclusions are as follows.

First of all, under the condition of emotional interference, bilingual experience affects executive function, but only in the early attention stage, which is consistent with the results of the N1 component of the ERP experiment. This shows explicitly that bilingual experience can improve an individual in attentional control ability and speed up attention processing;

Secondly, the effect of bilingual cognitive advantage is hindered or interfered with under emotional interference, which takes time to reflect in late task processing. The results of the P 3 component in the behavioral experiment and ERP experiments support this conclusion, manifested in the lack of significant differences in the RTs of task completion.

Thirdly, the interference effect of positive emotions is the most obvious among the three emotional conditions. Moreover, positive emotions can promote inhibition control and hinder switching ability as well as the updating function of working memory to a certain extent.

## Data availability statement

The raw data supporting the conclusions of this article will be made available by the authors, without undue reservation.

## Ethics statement

The studies involving human participants were reviewed and approved by the Ethics Committee of Yunnan Normal University. The patients/participants provided their written informed consent to participate in this study.

## Author contributions

YT: supervision, research design, writing, writing review and editing, project administration and crucial intellectual content revising. ZZ: conceptualization, research design, experiment instruction and implementation, data curation, formal analysis, investigation, methodology, resources and crucial intellectual content revising. YL: writing and experimenting comments, writing polishing and crucial intellectual content revising. All authors contributed to the article and approved the submitted version.

## Funding

This work was supported by the National Natural Science Foundation of China (Grant No. 32260205) and the National Social Science Fund of China (Grant No. 18XYY013).

## Conflict of interest

The authors declare that the research was conducted in the absence of any commercial or financial relationships that could be construed as a potential conflict of interest.

## Publisher’s note

All claims expressed in this article are solely those of the authors and do not necessarily represent those of their affiliated organizations, or those of the publisher, the editors and the reviewers. Any product that may be evaluated in this article, or claim that may be made by its manufacturer, is not guaranteed or endorsed by the publisher.
